# Mean platelet volume and mean platelet volume/platelet count ratio in risk stratification of pulmonary embolism

**DOI:** 10.1186/cc14408

**Published:** 2015-03-16

**Authors:** T Yardan, M Meric, C Kati, Y Celenk, A Atici

**Affiliations:** 1 Ondokuz Mayis University School of Medicine, Samsun, Turkey; 2 Ministry of Health Van Education & Research Hospital, Van, Turkey

## Introduction

Recently, mean platelet volume (MPV) was reported to predict early death in acute pulmonary embolism (PE). The aim of this study was to investigate the role of MPV and MPV/platelet count ratio (MPV/P) in risk stratification of patients with acute PE.

## Methods

We retrospectively reviewed the medical records of patients with acute PE admitted to the emergency department. In addition to the clinical evaluation, platelet count and MPV were measured on admission.

## Results

One hundred and fifty-two patients were included. Patients with right ventricular (RV) dysfunction had significantly higher MPV levels and MPV/P than patients without RV dysfunction. Receiver operating characteristic analysis revealed that a MPV cutoff of 7.85 flprovided 53.3% sensitivity and 68.5% specificity, and a MPV/P cutoff of 0.0339 fl/(10^9^/l) provided 69.6% sensitivity and 65% specificity for prediction of RV dysfunction. There was a positive correlation between MPV and systolic pulmonary artery pressure (SPAP) and between MPV and RV diameter. There was a positive correlation between MPV/P and SPAP and between MPV/P and RV diameter. The low-risk PE group had lower MPV and MPV/P than the massive PE and submassive PE groups.

## Conclusion

MPV and MPV/P are associated with RV dysfunction and clinical severity in acute PE. Low MPV and MPV/P levels may be an indicator of low risk in patients with acute PE.

